# A Case of Bilateral Acute Calcific Tendinitis of the Gluteus Medius, Treated by Ultrasound-guided Needle Lavage and Corticosteroid Injection

**DOI:** 10.5334/jbr-btr.810

**Published:** 2015-12-30

**Authors:** Elke Vereecke, Koen Mermuys, Jan Casselman

**Affiliations:** 1 UZ Gent, Ghent, BE; 2 AZ Sint-Jan AV Hospital, Bruges, BE

**Keywords:** calcific tendinitis, gluteus medius, calcium hydroxyapatite deposition disease, ultrasound guided needle lavage, local injection therapy

## Abstract

Calcium hydroxyapatite deposition disease is a common pathology, most frequently located in the rotator cuff tendons of the shoulder, for which different therapeutic approaches are used. Ultrasound guided needle lavage and injection of anesthetic/corticosteroid is a well-known and extensively described treatment for calcific tendinits of the rotator cuff. We present a case of bilateral calcific tendinitis of the gluteus medius tendon, both sides successfully treated using ultrasound guided needle lavage of the deposits and injection of an anesthetic and corticosteroid. We propose to not only use this approach for rotator cuff tendons, but also for calcific tendinitis at other locations.

## Case report

A 46-year-old woman presented at our institution in April 2013 with acute pain in the left hip. She had a skiing accident, being hit by another skier. Since then, she experienced severe persistent pain in the left hip. The medical history included few relevant findings, except bilateral recurrent calcific tendinitis of the rotator cuff, treated with corticosteroid injections, physiotherapy and arthroscopic exploration (with debridement of the calcific deposition, bursectomy and decompression of the subacromial space). Clinical examination showed pain and significant tenderness of the trochanteric region. There was a normal, however painful, range of motion.

**Figure 1:**
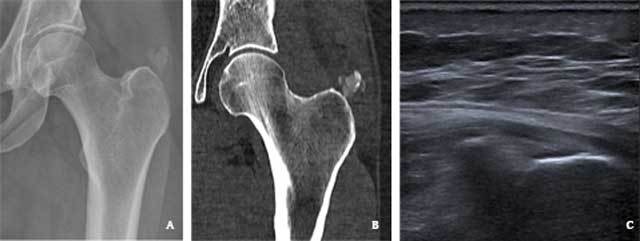
A 46-year-old woman with calcific tendinitis of the left gluteus medius tendon. (A) Plain radiography reveals a large calcification superior to the greater trochanter (B) CT with soft tissue window confirms this calcification (arrow), and locates it in the gluteus medius tendon. (C) Ultrasound image confirms a hyperechogenic calcification in the gluteus medius tendon.

A plain radiography excluded fractures, but demonstrated the presence of perithrochanteric calcifications (Fig. 1). A CT-scan of the pelvis confirmed the absence of fractures, and the presence of a large, well-defined calcification of low density, 18 millimeters in diameter, located anteriorly in the gluteus medius tendon (Fig. 1). Acute calcific tendinitis of the gluteus medius tendon was suggested as the cause of the patients’ pain.

Since ultrasound-guided needle lavage is a well-described therapy for hydroxyapatite depositions in the rotator cuff tendons of the shoulder, we proposed this treatment approach for this patients’ calcific tendinits. An ultrasonography was performed to assess the feasibility. The calcification was demonstrated as a hyperechogenic structure in the gluteus medius tendon within reach of a needle (3,1 cm beneath the skin) (Fig. 1). Reactive thickening of the overlying bursa was also seen. In consultation with the patient and the orthopedic surgeon, an ultrasound-guided needle lavage and corticosteroid injection was performed. Linisol 2% was used as local anesthetic in the subcutaneous tissue, in the overlying bursa and in the peritendinous tissue around the calcification. This reduced the pain instantaneously. A 21 Gauge long spinal needle was then inserted into the hydroxyapatite deposition. The deposition was rinsed using small syringes of linisol 1%, which were gently and alternatingly injected and aspirated. There was progressive aspiration of a large amount of white crystals into the syringes. Once no more crystals could be aspirated, the region of the calcification and the overlying bursa was infiltrated with 40 milligrams of depoMedrol, dissolved in 4 milliliters of marcaine 0,5%. The patient’s symptoms resolved within a few days following the procedure, and she could quickly resume her daily activities. No follow-up imaging was performed given the good response.

18 months later, in November 2014, the patient returned to the hospital with similar complaints, now in the right hip. The pain had an acute but non-traumatic onset. The orthopedic surgeon clinically suspected bursitis, and requested a magnetic resonance imaging-study (MRI) to confirm this hypothesis and to exclude intra-articular pathology.

**Figure 2:**
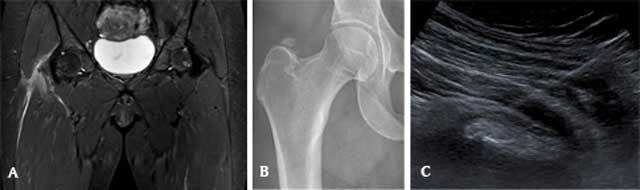
A 47-year-old woman with calcific tendinits of the right gluteus medius tendon. (A) Coronal Short Tau Inversion Recovery MRI image reveals extensive soft tissue edema (arrow) near the greater trochanter, centered around a hypo-intense calcification in the gluteus medius tendon, (B) plain radiography of the right hip confirms the calcification (arrow) in the soft tissue superior to the greater trochanter, (C) ultrasound image demonstrating the needle approaching the calcification.

The MRI showed extensive edema and infiltration of the soft tissue around the greater trochanter, between the proximal muscle heads of the quadriceps, and along the tensor fasciae latae muscle and around the insertion of the gluteus medius and minimus (Fig. 2). A hypo-intense, sharply defined structure with a length of almost 15 millimeters and a thickness of 8 millimeters, was identified in the gluteus medius tendon. There were no abnormalities of the joint. Due to the non-traumatic onset of the pain, a rupture or elongation of the vastus intermedius muscle of the quadriceps was unlikely. Because of the history of acute calcific tendinitis of the gluteus medius tendon on the left side, we interpreted the hypo-intese intratendineous structure as a calcification, with calcific tendinitis with manifest reactive edema as the most probable diagnosis. A radiography of the right hip confirmed a large, well-defined calcification cranial to the greater trochanter (Fig. 2).

An ultrasound-guided needle lavage of the calcification with corticosteroid injection was performed, in consultation with the referring orthopedic surgeon and the patient (Fig. 2). This procedure was carried out the same way as 18 months earlier on the left side. There was a similar good clinical response to the procedure (i.e. nearly complete alleviation of the pain within days and eventually a complete pain-free state), so no follow-up imaging was performed.

## Discussion

Calcium hydroxyapatite deposition disease is a well-known and well-described pathology. It is defined as deposits of calcium hydroxyapatite peri- or intra-articular [6]. When located in a tendon, it is also known as calcific tendinits. The shoulder is the most common location of calcium hydroxyapatite depositions, and the most frequently affected tendon is the supraspinatus tendon [5]. However, they may arise virtually anywhere. Cases of calcific tendinits have been described in the elbow, wrist, hand, hip, knee, ankle, foot, neck, … [4,5].

The diagnosis is often clinically suspected and confirmed on medical imaging. Plain radiography usually shows the presence of calcifications in the para-articular soft tissues [6]. These appear to be curvilinear, ill-defined globular or aciniform [3]. CT-scan has the advantage of better contrast resolution and absence of superposition, so it can be used to detect small calcifications which are sometimes invisible on radiographs, and to identify the precise localization in a tendon, ligament, … [3]. Sometimes aggressive features are recognized, such as cortical erosions [5]. Ultrasound has a great contrast and spatial resolution as well, and demonstrates the hydroxyapatite deposits as hyperechogenic masses, sometimes with acoustic shadowing [3,6]. It achieves this without irradiation. MRI does not imply any radiation dose either, and demonstrates edema (T1 hypointensity and T2 hyperintensity) around the calcifications and occasionally hydrops of the bursa [3,6]. The hydroxyapatite deposits are hypointens on T1- and T2-weighted images, and may lead to blooming artifacts on T2* gradient-echo sequences due to susceptibility, which makes this a very sensitive sequence for detection of hydroxyapatite [6].

Clinically, patients with calcific tendinitis of the gluteus medius present with pain at the anterolateral or posterolateral thigh, the buttock or lower back. Sometimes they have a decreased range of motion at the hip, or may experience difficulty in walking. Usually, there is extreme tenderness over the greater trochanter [3]. Calcific tendinitis should be considered in patients presenting with these symptoms.

Multiple case reports of calcific tendinitis of the gluteus medius tendon illustrate the usually self-limiting nature of the disease [3,4]. Therefore, similar to calcific tendinitis of the rotator cuff, initial treatment consists of conservative management, including immobilization, non-steroidal anti-inflammatory drugs and physical therapy. However, the symptoms can be rather severe and persistent, in which case a more aggressive approach can be considered, including needle aspiration and steroid injection, extracorporeal shockwave lithotripsy or surgery [4,5,6]. Ultrasound guided needle lavage of hydroxyapatite depositions and injection of anesthetic and/or corticosteroid is a well-know and well-described treatment for calcific tendinitis of the rotator cuff [1,8]. However, needle lavage therapy has only sporadically been described in other locations [1,5]. Moreover, numerous cases of calcific tendinitis of the gluteus medius and other tendons of the hip have been reported, but only few described local injection therapy of the gluteus medius tendon [2,4,5,7].

Yoo et al. performed ultrasound-guided needle decompression and subacromial corticosteroid injection on 30 patients with painful calcific tendinitis of the shoulder [8]. They found it to be effective within 6 months in 71,4% of the patients, with reduction of calcium deposit size, and with a mean time of 2,7 months to reach improvement of pain [8]. However, they achieved successful aspiration in only 6 out of 30 patients and no calcific material could be aspirated in 19 out of 30 patients. On the other hand, decompression was performed in all patients by multiple puncturing, suggesting the importance of decompression, rather than aspiration [8].

De Zordo et al. performed a retrospective study of ultrasound-guided perforation and lavage with corticosteroid injection in 40 patients with symptomatic calcific tendinitis of the rotator cuff tendons (34 patients), triceps, common flexor, common extensor, patellar and rectus femoris tendon [1]. They found a mean clinical improvement of 8.1/10 in the shoulder patients and 7.5/10 in non-shoulder patients, and identified only one complication (partial tear of a rotator cuff tendon). The authors concluded this treatment to be successful and safe in shoulder and non-shoulder calcific tendinitis [1].

Regarding calcific tendinitis of the gluteus medius, previously reported successful local injection therapy include direct local injection of anesthetic and corticosteroid [2,7] and ultrasound guided infiltration with anesthetic and steroid, combined with puncturing of the calcification [4]. Siegal et al. used ultrasound guided lavage and aspiration, but did not specify if the peritrochanteric calcification they treated is located in the gluteus maximus, medius or minimus [5]. Park et al. studied 30 hips in 29 patients with acute calcific tendinitis in the region of the hip (15 among them located in the gluteus medius tendon, others in the rectus femoris, the capsule, the piriformis and iliopsoas) [4]. Conservative treatment with non-steroidal anti-inflammatory drugs and analgesics was sufficient in 23 patients (24 hips), demonstrating once more the self-limiting nature of the disease. Two patients (one gluteus medius and one piriformis muscle) with extreme, nonresponsive pain were successfully treated with ultrasound guided local anesthetic and steroid injection, resulting in a rapidly diminishing of the pain and ultimately a pain-free state after 4 weeks [4]. Four other patients (2 gluteus medius and 2 capsula) with persistent pain after 3 months underwent arthroscopic excision, resulting in a pain-free status after 3 months [4]. This suggests more rapid response to injection of anesthetics and steroids, as opposed to surgical approach. There are currently no studies available comparing anesthetic/corticosteroid injection, with or without aspiration of calcium crystals and/or barbotage of the calcium deposit for calcific tendinitis of the gluteus medius.

In conclusion, ultrasound-guided needle lavage and injection of anesthetics and corticosteroids for gluteus medius calcific tendinitis appears to be effective, is minimally invasive, widely available, inexpensive and non-irradiating, as demonstrated by this case report and previous reports [4]. Therefore use of this treatment is proposed for calcific tendinitis of the gluteus medius tendon, and, by extension, for calcific tendinitis of other non-shoulder tendons.

## Competing Interests

The authors declare that they have no competing interests.
